# Miscellaneous Facts and Remarks

**Published:** 1799-06

**Authors:** 


### *Miscellaneous Facts and Remarks.*

IN the "*Recueil Periodique*" &c. No. XXVIII, we find a striking difference of opininion existing between two eminent practitioners, BAUDELOCQUE and LAUMONIER, respecting the *amputation of the uterus.*

The following is the observation of Cit. Laumonier: "On passing the finger under the urinary canal, on the anterior part of the vagina, there was found a gangrenous spot of about one inch square The operation is simple; I make a circular incision *above* the gangrenous spot," &c.

On this treatment Cit. Baudelocque makes the following remark: "A circular incision is first made in the neck of the tumour, *under* the gangrenous spot of which Cit. Laumonier speaks, and consequently near two inches under the *meatus urinalis,*" &c.

---

IN *apoplectic cases of children,* accompanied with fulness of the præcordia, and other symptoms of plethora, Prof. HUFELAND strongly recommends bleeding combined with emitics, in quick succession. The former facilitates the operation of the latter; prevents the danger which they might otherwise occasion; produces an antispasmodic effect; and disposes the emetics to remove the material cause, with greater safety and efficacy, from having an obvious tendency to produce the necessary change in the tone of the whole nervous system.

The indications for bleeding, in such instances, cannot be determined by the age of the patient, but by the urgency or danger of the case, the general constitution, the state of the pulse, and other circumstances.

In the case of a phlethoric child, six years of age, who had fallen into an apopleptic fit, Prof. Hufeland directed four ounces of blood to be drawn from the arm; and as a solution of four ounces of antim. tart. gradually given, produced no effect, prescribed three grains of white vitriol to be dissolved in one ounce and a half of water, of which a table spoonful was administered every fifteen minutes. This soon operated; the patient vomited a great quantity of mucus and other crude matter; became perfectly sensible; recovered his speech and respiration; the violence of the pulse and febrile heat abated; the convulsions ceased; and the patient completely recovered.

---

THE danger which always attends the opening of tumours of the knee, commonly called *white swellings,* is well known among practitioners, and and that there is scarcely any chance left for the unhappy sufferer, but amputation of the whole leg. Yet there are a few cases on record, in which emetics have proved of singular advantage in white swellings, upon the same principle as, according to the report of *Riverius*, they have been once observed to cure the hydrocele.

Prof. HUFELAND, in his valuable "Remarks on the inoculated Small-pox, and other diseases of children;" p. 582, relates the following extraordinary case:

"A youth, 14 years of age, was for a considerable time afflicted with a swelling of the knee, so that he at length was altogether prevented from using the leg in walking. For some other cause, he was obliged to take an emetic, which apparently produced a diminution of the swelling. In consequence of this change, I directed him to continue the use of the antimonial tartar, in small doses, and after an interval of eight days, to take another strong emetic. On the day following, I perceived, with a degree of surprise, that the swelling had decreased half an inch. This method was, therefore, regularly pursued; the emetic was continued every eight days, and the swelling covered with the common plaister of pitch. The tumour gradually disappeared, and the patient was completely cured in six weeks."

---

IN our last Number, p. 279, we gave a short account of the *carbon* being used in America, as a remedy for habitual costiveness. As this substance, at least in a chemical sense, is possessed of great *antiseptic* powers; and as we are not acquainted with any particular remedy which, *a priori*, is better calculated to remove *bilious, acrid*, and other *morbid matters*, from the *primæ viæ*, where they frequently produce the most important and critical changes particularly in acute diseases; it appears deserving the attention of the practitioner, to ascertain whether a few doses of powdered carbon recently burnt, might not, in such cases, be attended with the most beneficial effects.

The *external use of carbon*, for instance, in the *hæmorrhoids*, has long been known to our predecessors; and we are induced to quote the following curious passages from "*Hadriani à Mynsicht Thesauro & Armamentario Medico-Chemico*:" *Francofurti*, 1675, 8vo.—Sec. v. p. 133.

"*Pulvis de Verbasco*: Rj. Herb. verbasc. viridis q. v. infer crucibulo quantum capit, ad summum usque infarciendo, deinde alio contege crucibulo, luto bene muni, igni impone, ut nigrescat eo, non vero in cineres abeat, cumque videbitur satis esse, crucibulum fac refrigescere, atram illam materiam exime, & in subtilem pulverem redige. Postea,

Rj. *Hujus pulveris nigri,* unc. i. *Rhabarbari electi,* drach. ii. Mice & f. pulvis subtilissimus.

" *Vires & usus.* Certum hic pulvis est experimentum contra condylomata & hæmorrhoides caecas. Cujus usus est, ut aegrotus de *indusio viri trito* sumat particulam, eamque in uno latere paululum propria sua saliva humectet, de pulvere hoc aspergat, & ita superimponat toties repetendo quoties opus, donec condylomata & haemorrhoides illæ caecæ disspareant, quod brevi tempore evenit.

---

Typhus, particularly if attended with a putrid diathesis, is one of those diseases in the cure of which the whole Materia Medica is frequently ransacked, without enabling us to afford much relief to the afflicted. If, what a practitioner in Germany, Dr. Krügelstein, has asserted with much confidence, in a late publication on that subject, be true, we shall henceforth be enabled to cure a putrid fever as readily as an ague, and perhaps with more expedition than consumption is overcome by the digitalis.

According to the experience of Dr. K. the malignancy of *typhus gravior* has in many cases yielded to *strong doses of mineral and vegetable acids,* when given alternately in such a manner, that the effect of the succeeding dose may begin, before that of the preceding one has entirely vanished. He goes still farther, and maintains that even pestilential disorders may be overcome by the combined powers of these acids. His method of exhibiting them is as follows: he first gives a dose of cream of tartar (from one to two drachms, according to the circumstances of the case), and immediately after it, from five to fifteen drops of vitriolic acid diluted with a sufficient quantity of water. These draughts are successively administered to some patients every hour, to others every two, three, or four hours. Dr. K. relates wonderful effects produced by this new practice, but we have some reason to apprehend, he has mistaken the nature of the fever. Vid. *Journal of Inventions,* No. I. p. 131.

---

As the removal of *biliary calculi* has hitherto been considered impracticable by any remedies taken into the stomach, our readers may not, perhaps, in general be acquainted with a remedy proposed for that purpose by a late French writer, M. Durand. In hepatic complaints arising from biliary concretions, he prescribes from ten to twenty drops of a mixture consisting of equal parts of vitriolic ether and spirit of turpentine, to be taken several times in the course of the day, assisted by opium, laxatives, emollient clysters, &c. according to the circumstances of the case.

